# Representation of complex vocalizations in the Lusitanian toadfish auditory system: evidence of fine temporal, frequency and amplitude discrimination

**DOI:** 10.1098/rspb.2010.1376

**Published:** 2010-09-22

**Authors:** Raquel O. Vasconcelos, Paulo J. Fonseca, M. Clara P. Amorim, Friedrich Ladich

**Affiliations:** 1 Departamento de Biologia Animal, Centro de Biologia Ambiental, Faculdade de Ciências, Universidade de Lisboa, Bloco C2 Campo Grande, 1749-016 Lisbon, Portugal; 2 Unidade de Investigação em Eco-Etologia, I.S.P.A. Rua Jardim do Tabaco 34, 1149-041 Lisbon, Portugal; 3 Department of Behavioural Biology, University of Vienna, Althanstrasse 14, 1090 Vienna, Austria

**Keywords:** hearing, temporal encoding, amplitude modulation detection, auditory evoked potential, conspecific sounds, Batrachoididae

## Abstract

Many fishes rely on their auditory skills to interpret crucial information about predators and prey, and to communicate intraspecifically. Few studies, however, have examined how complex natural sounds are perceived in fishes. We investigated the representation of conspecific mating and agonistic calls in the auditory system of the Lusitanian toadfish *Halobatrachus didactylus*, and analysed auditory responses to heterospecific signals from ecologically relevant species: a sympatric vocal fish (meagre *Argyrosomus regius*) and a potential predator (dolphin *Tursiops truncatus*). Using auditory evoked potential (AEP) recordings, we showed that both sexes can resolve fine features of conspecific calls. The toadfish auditory system was most sensitive to frequencies well represented in the conspecific vocalizations (namely the mating boatwhistle), and revealed a fine representation of duration and pulsed structure of agonistic and mating calls. Stimuli and corresponding AEP amplitudes were highly correlated, indicating an accurate encoding of amplitude modulation. Moreover, Lusitanian toadfish were able to detect *T. truncatus* foraging sounds and *A. regius* calls, although at higher amplitudes. We provide strong evidence that the auditory system of a vocal fish, lacking accessory hearing structures, is capable of resolving fine features of complex vocalizations that are probably important for intraspecific communication and other relevant stimuli from the auditory scene.

## 1. Introduction

Fishes depend on their auditory system to interpret information from the acoustic environment, including predator and prey detection (e.g. [1]), and to communicate acoustically. Many teleosts have evolved a variety of sound-producing mechanisms and vocalizations that are crucial not only for mate attraction but also during social interactions [2,3]. Temporal characteristics of sounds are thought to be the most important for acoustic communication in fishes because most calls consist of series of short broad-band pulses (e.g. gudgeons, gouramis, catfishes [4,5]). Sound variability, however, also relies on other differences, such as dominant frequency, and less commonly on frequency and amplitude modulation [6]. This variability plays a role in the social life of fishes by providing information to assess the size of the calling individual (e.g. dominant frequency [7,8]), to identify motivation for mating (e.g. calling rate [9,10]) and to recognize conspecifics from other vocally closely related species (e.g. [11–13]). Behavioural observations have shown that fishes can respond selectively to acoustic stimuli varying in temporal patterns and frequency content. Playback experiments with the toadfish *Opsanus tau* and the midshipman fish *Porichthys notatus* (Batrachoididae) demonstrated that males may alter their own calling rate in response to another male calling [14] and that females move towards the sound source depending on the signal temporal content, frequency and amplitude (including modulation) [15]. Temporal patterns, frequency and amplitude modulation of sounds are clearly important for acoustic communication in other taxa such as insects, anurans, birds and mammals (e.g. [16–20]).

Most studies on fish audition have used artificial stimuli to test hearing abilities (e.g. [12,21–26]). Accordingly, the representation of complex conspecific sounds in a fish's auditory system remains almost uninvestigated. To date, only two studies have examined how conspecific sounds (mostly short and pulsed calls) are represented in the auditory pathway. Wysocki & Ladich [27] analysed auditory evoked potentials (AEPs) in response to conspecific sounds in fishes possessing accessory morphological structures for enhancing hearing sensitivity (catfishes *Platydoras costatus* and *Pimelodus pictus*, loach *Botia modesta* and gourami *Trichopsis vittata*) and in a species lacking specializations, the sunfish *Lepomis gibbosus*. Species possessing hearing specializations generally showed an accurate representation of sound temporal patterns, amplitude fluctuations and, solely in *P. pictus*, a clear representation of the harmonics of its drumming sounds. In contrast, *L. gibbosus* did not exhibit an accurate temporal or frequency resolution. More recently, Maruska & Tricas [28] analysed the response properties of central auditory neurons to conspecific signals in a species without specializations, the damselfish *Abudefduf abdominalis*. The authors found that thresholds were lower in the midbrain than the hindbrain, and that the species was most sensitive to the frequency and temporal components of its natural pulsed calls.

Representatives of the family Batrachoididae (Teleostei, Actinopterygii), which include toadfishes and the plainfin midshipman fish, have emerged as one of the main study models for both behavioural and neurobiological studies in fish acoustic communication [29]. The rich vocal repertoire in this group is rare among fishes and includes long tonal advertising sounds. This suggests that the sensory system of batrachoidids is probably adapted to encode different sound characteristics of communication signals. The present study was designed to investigate the representation of complex conspecific sounds in the auditory system of the Lusitanian toadfish *Halobatrachus didactylus* (Bloch & Schneider 1801). This species produces at least five different vocalizations [13,30] and some sound characteristics, such as pulse interval, dominant frequency and amplitude of the agonistic calls, are correlated with fish size [31]. Besides, the complex amplitude-modulated advertising sounds (boatwhistles) reveal individual differences that may provide cues for mate choice and assessment of opponents [32].

Our primary goal was to investigate whether temporal patterns, amplitude modulation and frequency content of agonistic grunts and mating boatwhistles are encoded by the toadfish auditory brainstem. We also analysed the auditory responses to sounds from two ecologically relevant species—a sympatric vocal fish (meagre *Argyrosomus regius*) and a potential predator (bottlenose dolphin *Tursiops truncatus* [1])—in order to evaluate the extent to which this species is adapted to interpret other relevant information from its acoustic environment.

## 2. Material and methods

### (a) Animals

The test subjects were 16 Lusitanian toadfish: eight females (23–28 cm total length, TL) and eight type I males (25–32 cm TL), caught in the Tagus estuary (Portugal) during the breeding season (late June) by local fishermen. Fish were kept in two 250 l tanks for two weeks prior to the auditory recordings. Females were identified by their larger abdomens and/or their wider genital papilla. Type I males, which possess smaller gonads but larger accessory glands and more developed sonic muscles [33], were recognized by the secretion of their accessory glands.

### (b) Auditory evoked potential recording setup

The AEP technique is a non-invasive method that records the overall synchronous neural electric activity induced by acoustic stimulation [34] and proved to be valuable for studying the perception of conspecific vocalizations (e.g. porpoises [35]; teleost fishes [27]). Test subjects were mildly immobilized with Flaxedil (gallamine triethiodide; Sigma-Aldrich, Austria; dosage 4.8–15.0 µg g^−1^) and placed just below the water surface in an oval-shaped plastic tub (diameter 45 × 30 cm; water depth 12 cm) with the exception of the electrode contacting points. Fish respiration was secured through a simple temperature-controlled (21 ± 1°C) gravity-fed water system using a pipette inserted into the subject's mouth. The recording electrode was located above the brainstem and the reference electrode approximately 2 cm rostrally (silver wire, 0.25 mm diameter). Shielded electrode leads were attached to the differential input of an AC preamplifier (Grass P-55, Grass Instruments, USA; gain 100×, high-pass at 30 Hz, low-pass at 1 kHz). A ground electrode was placed in the water near the fish body. A hydrophone (Brüel and Kjaer 8101, Denmark; voltage sensitivity –184 dB re 1 V µPa^−1^) was placed on the right side of the subject near the inner ear (approx. 2 cm away) in order to control for stimulus characteristics. The experimental tub was positioned on an air table (TMC Micro-g 63–540, Technical Manufacturing Corporation, USA) inside a walk-in soundproof room. Both sound stimuli and AEP waveforms were recorded using a Tucker-Davis Technologies (USA) modular rack-mount system (TDT System 3) controlled by a computer containing a TDT digital processing board and running TDT BioSig RP software.

### (c) Sound stimuli presentation

Two advertising boatwhistles with different dominant frequencies of 93 Hz (bw1) and 44 Hz (bw2), produced by nesting toadfish males (35–48 cm; 963–1819 g) in the Tagus estuary (Portugal), were chosen among previously field-recorded sounds [36]. An agonistic grunt train composed of three grunts recorded from an adult female (25.0 cm TL, 492 g) and a single grunt produced by a juvenile (10.5 cm TL, 15.5 g) was also selected from previous laboratory recordings [31]. To test for temporal encoding, we also considered two other modified boatwhistles created from the original bw1 that was shortened by 149 ms (bw1short) or extended by 298 ms (bw1long) in the middle of the tonal phase. An additional sound presentation consisted of two boatwhistles emitted in sequence (i.e. bw1 followed by bw2 after a 50 ms interval), mimicking two vocalizing male neighbours.

Heterospecific calls consisted of a segment of a sequence of pops produced by a bottlenose dolphin *T. truncatus* during conspecific social interactions and foraging in the Sado estuary, provided by M. E. dos Santos. The bottlenose dolphin has been described as a potential predator of batrachoidids [1], including *H. didactylus* in Sado River, Portugal [37]. We also considered a mate advertising call emitted by a male meagre *A. regius* (Sciaenidae) previously recorded in the Guadiana River, Portugal (N. Prista & M. C. P. Amorim). Breeding meagre males are relatively large (up to 2 m long), emit high-amplitude long tonal calls (probably used for mate attraction [38]), and inhabit the coastal areas in the eastern Atlantic and Mediterranean where Lusitanian toadfish breeding aggregations are also found (e.g. Tagus River; R. O. Vasconcelos 2006–2008, personal observations).

Sound wave stimuli files (25 kHz sampling frequency) were imported into TDT SigGen 3.2 software and fed through a real-time processor (RP 2.1) into a programmable attenuator (PM 5). Two speakers including a sub-woofer (Fostex PM-0.5 Sub and PM-0.5 MKII, Fostex Corporation, Japan) were positioned 50 cm above the experimental tub and used to play back sounds. Stimuli repetition rate varied from 0.8 to 2.7 per second. Each stimulus was presented at least 500 times at opposite polarities and the two AEP traces obtained were then averaged. This procedure using natural sounds at opposite polarities efficiently eliminated eventual stimulus artefacts in the AEPs recorded in our setup because auditory responses are not affected by polarity changes [27]. Sound pressure levels (SPLs) used were monitored with a hydrophone (Brüel and Kjaer 8101) connected to the sound level meter (Brüel and Kjaer 2238 Mediator). Sounds were first presented at 123–129 dB re 1 µPa (depending on the stimulus), and then attenuated in 4 dB steps until recognizable and repeatable auditory response could no longer be detected. The lowest SPL at which a repeatable AEP trace correspondent to specific sound pulses could be obtained, as determined by overlaying replicate traces, was considered the threshold. This method of visual inspection/correlation of hearing thresholds has been traditionally used in AEP audiometry [27,34].

Toadfishes possess no known hearing specialization and are most probably sensitive to particle motion [39,40]. We therefore provide hearing thresholds in sound pressure and particle acceleration levels. For this purpose, a calibrated underwater miniature acoustic pressure–particle acceleration (p–a) sensor S/N 2007-001 (Applied Physical Sciences Corp., Groton, CT, USA) was placed at the fish's position in the test tub. Particle acceleration levels (*L*_a_) were determined for all sound stimuli at various levels, including the hearing threshold levels of the species, with the acceleration sensor oriented in all three orthogonal directions. Similar to Wysocki *et al*. [41], the total acceleration level was calculated based on the acceleration level of each axis in micrometers per second square as 20 log(√(*x*^2^ + *y*^2^ + *z*^2^)). Pressure and particle acceleration were positively correlated to each other below the water surface in the experimental tub, and any 4 dB change in SPL was generally accompanied by a 4 dB change in particle acceleration level for all stimuli.

### (d) Auditory response waveform analysis and statistics

Detailed waveform and spectral analysis were performed using Audition 2.0 (Adobe Systems Inc., CA, USA) and Raven 1.2 (Cornell Laboratory of Ornithology, NY, USA) at the maximum amplitude tested (123–129 dB re 1 µPa 84–92 dB re 1 µm s^−2^, approx. 14–31 dB above hearing thresholds, depending on the stimuli). Stimuli and AEP durations were determined to evaluate temporal resolution. The onset of the auditory response was considered the beginning of the first positive peak, which was typically delayed by approximately 7–11 ms relative to the onset of the sound stimulus. The end of the AEP trace was considered the last peak clearly distinguished from the ongoing noise.

Spectral peaks of sound and corresponding AEP (sampling frequency 20 kHz, 8192 points FFT size, Hamming window) were compared to verify whether the main frequency content of sounds was represented within the auditory response [27,42].

To evaluate the representation of the boatwhistles' amplitude modulation (bw1, bwshort, bwlong, bw1 + bw2) in the auditory responses, the envelopes of both acoustic stimuli and AEPs were compared. Stimuli and response envelopes were extracted by calculating a moving average of maximum amplitude values of the waveforms using a moving window of 7 ms. The choice of window length is critical and in this case 7 ms was used as a compromise between the period at 93 Hz (stimulus dominant frequency) and the period expected if a double frequency occurs in the AEP response. The stimulus and corresponding AEP envelopes with the same duration or number of points (21 484–47 606 points) were compared using Pearson's correlations. This method was validated by correlating the envelopes of sound stimuli with envelopes of white noise sequences with the same duration (three different white noise sequences for each stimulus), but also by correlating the envelopes of boatwhistles of other toadfish (e.g. bw2, with different dominant frequency but similar amplitude modulation) with AEP responses to bw1. We also correlated the envelope of another mate advertisement boatwhistle (bw3) produced by a nesting toadfish male previously recorded in the Tagus estuary [36], with different dominant frequency (227 Hz) and amplitude modulation, with AEPs to bw1. This validation should produce low correlation coefficients in both cases, in contrast to the high coefficients expected for the stimulus versus corresponding AEP response.

Thresholds to all sound stimuli were compared with a one-way ANOVA performed with all data (from males and females) followed by a Bonferroni post hoc test to verify specific differences between sound stimuli. Mann–Whitney *U* tests were used to compare hearing thresholds to conspecific stimuli (bw1, bw2 and grunt train) between males and females.

Parametric tests were performed when data were normally distributed and variances were homogeneous. The statistical tests were run using Statistica 7.1 for Windows (StatSoft, Inc., USA).

## 3. Results

### (a) Representation of temporal patterns

**Figure 1.**
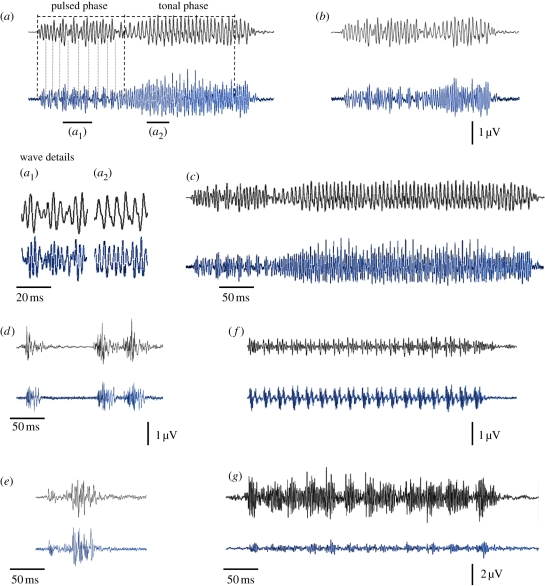
Oscillograms of each sound stimulus (upper black trace) and corresponding auditory evoked response (lower blue trace) recorded from *H. didactylus*. Sound stimuli shown consist of: (*a*–*c*) conspecific mate advertising boatwhistles ((*a*) original bw1; waveform details of the (*a*_1_) pulsed and (*a*_2_) tonal phase; and modified boatwhistles, (*b*) bw1short and (*c*) bw1long); (*d,e*) conspecific agonistic calls ((*d*) adult grunt train and (*e*) juvenile grunt); and (*f,g*) heterospecific sounds ((*f*) mate advertising call of meagre *A. regius* and (*g*) foraging pops of bottlenose dolphin *T. truncatus*). Averaged stimuli and AEPs depicted resulted from 1000 recordings in one specimen obtained at 123–129 dB re 1 mPa, approximately 14–31 dB above hearing thresholds depending on the stimuli. The amplitude of sound waveforms was adjusted to better fit AEP traces.

The temporal structure of conspecific sounds was accurately represented in the auditory responses in both males and females (*n* = 16 fish). Each sound pulse generally elicited a separate AEP waveform. Auditory responses to the boatwhistle bw1 showed a representation of both parts of the call, namely the pulsed part and the longer tonal part (see figure 1*a*, *a*_1_ and *a*_2_ for waveform details). Changes in the boatwhistle duration were accurately represented in the auditory system (figure 1*b*,*c* and table 1).

**Table 1.** Duration (ms) of sound stimuli and corresponding AEP responses (mean ± s.e. and range). bw1, original boatwhistle; bw1short, bw1 shortened in the tonal phase; bw1long, bw1 extended in the tonal phase; gr1–gr3, grunts emitted in a train by an adult; juv gr, juvenile grunt.

	mating boatwhistles			agonistic grunts			
	bw1	bw1short	bw1long	gr1	gr2	gr3	juv gr
stimulus	617	430	988	80	77	84	88
AEP	614 ± 2 (601–632)	439 ± 4 (421–477)	998 ± 3 (976–1015)	67 ± 1 (57–82)	74 ± 1 (65–80)	80 ± 2 (58–101)	85 ± 1 (80–89)

Agonistic grunt trains elicited AEP waveforms that corresponded exactly to each single grunt with similar durations and pulse structure (figure 1*d* and table 1). AEPs obtained in response to the juvenile grunt also revealed identical, precise temporal resolution (figure 1*e* and table 1).

Heterospecific sounds elicited AEP waves that generally followed the temporal patterns of the stimuli (figure 1*f*,*g*), although in most cases the waves could not be attributed to separate sound pulses, especially when responding to dolphin foraging pops. A clear auditory response was only verified at relatively high sound amplitudes, usually above 119 dB for the meagre advertising call and 124 dB for dolphin pops.

### (b) Representation of amplitude modulation

Amplitude modulation of conspecific boatwhistles was well represented in the auditory responses (figure 1*a*–*c*). The amplitude of these calls, represented by their envelope, was highly correlated with the amplitude of the AEP waveforms for all 16 specimens analysed: bw1 (*r* = 0.619–0.842, *p* < 0.001), bw1short (*r* = 0.556–0.780, *p* < 0.001) and bw1long (*r* = 0.654–0.785, *p* < 0.001). Moreover, amplitude values of the stimulus composed of two different boatwhistles (i.e. bw1 followed 50 ms after by bw2) were highly correlated with the amplitude values of AEPs (*r* = 0.517–0.691, *p* < 0.001). This indicated that the toadfish auditory system is capable of resolving amplitude fluctuations of different boatwhistles emitted sequentially. As expected, simulations with white noise (instead of AEP responses) and amplitude values of the different stimuli revealed no significant correlations: bw1 (*r* = −0.017–0.020, n.s.), bw1short (*r* = −0.115–0.032, n.s.), bw1long (*r* = −0.043–0.031, n.s.) and bw1 + bw2 (*r* = −0.068–0.045, n.s.). Moreover, correlations between bw2 and AEP to bw1 were not significant (*r* = −0.354–0.502, n.s.), nor were they between bw3 and AEP to bw1 (*r* = −0.029–0.082, n.s.).

### (c) Representation of frequency content

**Figure 2.**
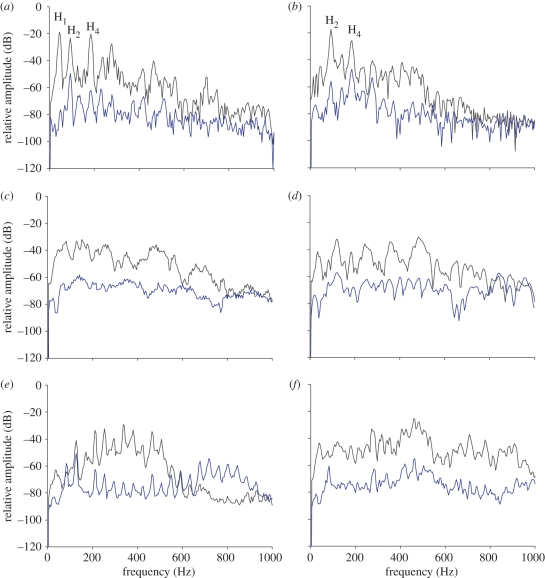
Power spectra of sound stimuli and AEP responses to conspecific mate advertising boatwhistles ((*a*) bw2 and (*b*) bw1), (conspecific agonistic sounds (*c*) adult grunt train and (*d*) juvenile grunt; and heterospecific sounds (*e*) mate advertising call of meagre *A. regius* and (*f*) foraging pops of bottlenose dolphin *T. truncatus*. Averaged stimuli and AEPs depicted resulted from 1000 recordings in one specimen obtained at 123–129 dB re 1 µPa, circa 14–31 dB above hearing thresholds depending on the stimuli. Sampling frequency 20 kHz, 4096 points FFT, 50% overlap, Hamming window. Black lines, sound stimulus; blue lines, AEP.

AEP waveforms evoked by bw1 and bw2 showed spectral peaks corresponding exactly to the several harmonics presented in the sound spectra (figure 2*a*,*b*). As expected, the dominant frequency of each AEP spectrum was typically twice the dominant frequency of the respective sound stimulus (table 2 and figure 2*a*,*b*).

**Table 2.** Dominant frequency (Hz) of sound stimuli and corresponding AEP responses (mean ± s.e. and range). bw1, bw2, boatwhistles; gr train, adult grunt train; juv gr, juvenile grunt; *Ar*, *A. regius*; *Tt*, *T. truncatus*. Sampling frequency 20 kHz, 8192 FFT size.

	conspecific sounds				heterospecific sounds	
	bw1	bw2	gr train	juv gr	*Ar* call	*Tt* pops
stimulus	93	44	151	481	339	461
AEP	180 ± 1 (173–183)	100 ± 6 (83–139)	141 ± 9 (93–225)	310 ± 71 (81–845)	300 ± 26 (127–381)	590 ± 95 (239–918)

The other conspecific agonistic sounds—adult grunt train and juvenile grunt—did not present a harmonic structure. Although the spectrum of AEPs showed some corresponding energy peaks to the sound spectrum, an association between both spectra was generally less clear (figure 2*c*,*d*). Dominant frequencies were more variable within AEPs obtained with these stimuli (table 2). Nevertheless, lower frequency adult grunts mainly generated AEPs with lower frequency energy than did higher frequency juvenile grunts. This suggests that the frequency content of agonistic sounds was also represented in the auditory system.

Heterospecific sounds exhibited relatively high dominant frequencies. The drumming sound of *A. regius* was harmonic, and a good match was observed between the AEP spectrum and each spectral peak of the sound stimulus. However, the dominant frequencies of both spectra differed considerably (figure 2*e* and table 2). AEPs elicited by *T. truncatus* foraging pops showed a general correspondence in some spectral peaks, but the main energy varied considerably (figure 2*f* and table 2).

### (d) Auditory sensitivity

**Figure 3.**
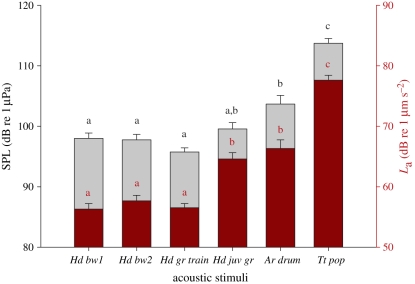
Mean (± s.e.) hearing thresholds, given as sound pressure levels (SPL, grey bars) and as particle acceleration levels (*L*_a_, dark red bars), to conspecific and heterospecific sounds. Conspecific stimuli (*Hd*, *H. didactylus*): *Hd bw1*, mate advertising boatwhistle with 93 Hz dominant frequency; *Hd bw2*, boatwhistle with 44 Hz dominant frequency; *Hd gr train*, agonistic adult grunt train; *Hd juv gr*, agonistic juvenile grunt. Heterospecific stimuli (*Ar*, *A. regius*; *Tt*, *T. truncatus*): *Ar drum*, mate advertising sound; *Tt pop*, foraging pop sound. Groups that are significantly different (*p* < 0.01) are indicated by different letters (results from Bonferroni post hoc tests).

Mean (±s.e., standard error) hearing thresholds for conspecific boatwhistles were 98.0 ± 0.9 dB re 1 µPa (56.3 ± 0.9 dB re 1 µm s^−2^) for bw1, 97.8 ± 0.9 dB re 1 µPa (57.7 ± 0.9 dB re 1 µm s^−2^) for bw2, 95.8 ± 0.7 dB re 1 µPa (56.5 ± 0.7 dB re 1 µm s^−2^) for adult grunt trains and 99.6 ± 1.0 dB re 1 µPa (64.6 ± 1.0 dB re 1 µm s^−2^) for juvenile grunts. Heterospecific calls evoked responses at higher levels: 103.7 ± 1.4 dB re 1 µPa (66.3 ± 1.4 dB re 1 µm s^−2^) for *A. regius* calls and 113 ± 0.8 dB re 1 µPa (77.6 ± 0.8 dB re 1 µm s^−2^) for *T. truncatus* pops. Thresholds varied significantly between sound stimuli (SPL: one-way ANOVA, *F*_5,70_ = 30.50, *p* < 0.001; *L*_a_: one-way ANOVA, *F*_5,70_ = 51.6, *p* < 0.001) and revealed significant differences (Bonferroni post hoc tests, *p* < 0.01) between conspecific and heterospecific calls. The exceptions were the toadfish juvenile grunt and the *A. regius* call (figure 3). Hearing thresholds (for bw1, bw2 and grunt train) did not differ between males and females (SPL, *L*_a_: Mann–Whitney *U* test, *U* = 22 − 29, *n*_1_ = *n*_2_ = 8, n.s).

## 4. Discussion

Most studies that have investigated the hearing abilities of fishes have used only artificial stimuli, such as pure tones [21–24,26], tone bursts [34,43,44] and clicks [12,25]. These studies have mostly aimed to describe species-specific audiograms, but also examine other aspects of auditory processing such as coding of temporal and intensity patterns, as well as spectral content. Some artificial stimuli approached the characteristics of conspecific calls [12,22], but did not fully reflect the overall complexity of vocalizations that animals produce and detect in their environment. To date, only two studies have analysed how conspecific sounds, mostly short-pulsed calls, are encoded by the auditory system in fishes [27,28]. The present study provides first data on the representation of complex conspecific vocalizations, including amplitude-modulated tonal calls, in the auditory system of a strongly vocal fish that lacks accessory hearing structures.

We showed that, in *H. didactylus*, both sexes can accurately resolve temporal patterns of conspecific signals. Auditory responses to the advertising boatwhistle showed a fine representation of each pulse and the distinct phases of the call (pulsed and tonal). Changes in boatwhistle duration were also accurately perceived. Agonistic grunts, including the juvenile call, were well encoded in their temporal characteristics (number of pulses, interval between grunts). The temporal pattern is thought to be the most important sound characteristic for acoustic communication in fishes, especially in noisy and/or shallow waters, where low frequencies do not propagate well and the spectral content of signals is easily altered [45]. Temporal information, such as the pulse period, seems to be important for intraspecific communication and species recognition (e.g. damselfishes [46]; gouramis [11]; electric fish [12]; cichlids [13]). According to Wysocki & Ladich [27], in the sunfish *L. gibbosus*, a species lacking accessory hearing structures, AEPs elicited by conspecific sound pulses were very long and did not follow specific pulses. In contrast, the results obtained with *H. didactylus* point to a fine temporal resolution comparable to those of species possessing hearing specializations (e.g. *P. pictus* and *T. vittata* [27]). This species exhibits an unusually complex acoustic repertoire that mostly varies in its temporal features (i.e. pulse interval, duration and repetition rate [13,30]). One of the parameters most probably used to distinguish between advertising nesting males is the boatwhistle duration and pulse period [32]. Moreover, other sound features such as repetition rate and duration of the agonistic grunts are correlated with fish size [31]. Therefore, detecting the temporal patterns of sounds is likely to be valuable for social interactions and mate attraction in *H. didactylus*. Previous behavioural studies reported that toadfishes (*O. tau* and *O. beta*) are able to produce an agonistic grunt on top of another toadfish's call after an average latency of 69 ms. This phenomenon (termed acoustic tagging) indicates a rapid response of the auditory component of a behavioural (sensory-motor) loop [47,48]. Our study confirmed that temporal patterns of both tonal advertising boatwhistles and pulsed agonistic grunts are precisely perceived and may help fish to extract important information during acoustic communication.

Amplitude modulation of advertising boatwhistles was also well represented in the auditory responses. Amplitudes of boatwhistles were highly correlated with the amplitudes of the auditory responses, independent of signal duration. A significant amplitude correlation was also found when two different boatwhistles were played back in sequence. This suggests that this parameter is well encoded even in the presence of more than one calling male, which typically occurs in toadfish breeding aggregations [32]. Marked amplitude modulation is found in boatwhistles produced by competing males in an advertising context. This contrasts with boatwhistles emitted during territorial defence, suggesting that this sound characteristic might be important for mate attraction but also informative of the social context in *H. didactylus* [36]. The perception of amplitude modulation has been poorly investigated in fish, probably because most species do not produce long amplitude-modulated sounds. Bodnar & Bass [22,24] investigated the neural responses in the batrachoidid *P. notatus* to simultaneous pure tones that form acoustic beats, similar to what occurs in a natural chorus. The authors found that midbrain units encode spectral and temporal features of concurrent signals (i.e. intensity and depth of modulation of beats).

We also showed that the frequency content of sounds, especially the multi-harmonic boatwhistles, can be perceived by *H. didactylus*. AEPs evoked by the boatwhistles showed spectral peaks corresponding exactly to the harmonics presented in the sound spectrum. The dominant frequency of the AEP spectrum was typically twice the dominant frequency of the corresponding sound stimulus. Such a frequency-doubling effect of AEPs, which is a further reassurance of a biological response, can be explained by the fact that saccular hair cells are oriented in opposite directions [49,50]. This phenomenon has also been observed in other fish species using the same AEP recording technique [51–53]. The frequency content of agonistic sounds was not as clearly represented in the auditory system, although a general match between the main energy of the stimulus and the AEP spectrum was detected, along with a distinct auditory response to juvenile and adult grunts. The dominant frequency of agonistic grunts is related to the body size in *H. didactylus* [31], similar to other teleosts [7,8]. Detection of the spectral content of vocalizations might be important in assessing the fighting ability of opponents and the quality of potential mates [8,54,55].

Hearing thresholds to conspecific signals did not differ between sexes in Lusitanian toadfish. Type I males nest in aggregations and vocalize in choruses to attract females. Behavioural evidence with this species showed that nesting males interact acoustically and alter their own boatwhistle calling rate in response to other calling males (J. M. Jordão, P. J. Fonseca & M. C. P. Amorim 2008, personal observations). These acoustic interactions suggest that the auditory system of nesting males must be adapted to detect and resolve acoustic parameters of boatwhistles similar to females, which probably select mates based on acoustic cues [15]. Hearing thresholds to higher-frequency heterospecific calls were higher than thresholds to conspecific signals, indicating that the Lusitanian toadfish is better adapted to detect intraspecific low-frequency vocalizations. Nevertheless, this species not only detected but also to some extent resolved temporal features of heterospecific sounds, namely of the advertising calls of the sympatric sciaenid *A. regius* and foraging sounds of *T. truncatus. Argyrosomus regius* inhabits coastal areas where Lusitanian toadfish breeding aggregations are usually found and produces advertising calls often at the same time (R. O. Vasconcelos 2006–2008, personal observations). Our results indicated that toadfish can discriminate between both conspecific and heterospecific multi-harmonic calls, in terms of temporal and amplitude patterns, and spectral content. The bottlenose dolphin *T. truncatus* has been described as a potential predator of batrachoidids [56], including *H. didactylus* [37]. Remage-Healey *et al*. [1] reported that playbacks of *T. truncatus* foraging pops considerably reduced the calling rate of the Gulf toadfish *O. beta* and induced an increment in cortisol levels. Our data indicate that the Lusitanian toadfish intercepts dolphin foraging sounds and support the previous behavioural observations.

In summary, we provide strong evidence that the auditory system of a highly vocal fish, lacking accessory hearing structures, can detect the fine temporal, amplitude and spectral features of complex vocalizations that are potentially important for acoustic communication. Future studies will determine the encoding properties of specific regions of the Lusitanian toadfish auditory system as AEP only reflects overall responses of the auditory pathway (saccule hair cells, eight nerve and brainstem auditory nuclei) up to the midbrain [57].
